# Trypanosomes of fish as an emerging threat to aquaculture systems in China

**DOI:** 10.1371/journal.ppat.1014129

**Published:** 2026-04-28

**Authors:** Ju-Feng Wang, Xin-Tao Li, Julius Lukeš, De-Hua Lai

**Affiliations:** 1 MOE Key Laboratory of Gene Function and Regulation, State Key Laboratory of Biocontrol and Guangdong Provincial Key Laboratory of Aquatic Economic Animals, School of Life Sciences, Sun Yat-Sen University, Guangzhou, P. R. China; 2 Institute of Parasitology, Biology Centre, Czech Academy of Sciences, and Faculty of Science, University of South Bohemia, České Budějovice (Budweis), Czech Republic; Boston Children’s Hospital, UNITED STATES OF AMERICA

## Introduction

Trypanosomes are prototypical haemoparasites capable of infecting a wide variety of vertebrate hosts [1]. Their life cycle stages occur mostly in the host’s bloodstream, as elongated, twisted cells with a prominent anterior flagellum [2]. The general strategy of well-established parasites is to multiply in their hosts without causing excessive or rapidly progressing pathology [3]. This is true also for the vast majority of infections caused by members of the genus *Trypanosoma*, despite notable reports of high pathogenicity [4]. Hence, most trypanosomiases are (very) mild and asymptomatic, with just one or a few flagellates found in the peripheral blood, as frequently documented in infected fishes, frogs, birds, and mammals [5–8]. However, the best studied trypanosomes are responsible for highly pathogenic or lethal infections, such as human African trypanosomiasis and nagana of cattle caused by *T. brucei rhodesiense* and *T. b. brucei*, respectively [9], with serious pathology most likely caused by the relatively recent acquisition of these hosts in evolutionary terms [10].

So far, trypanosomes infecting fish belonged to a category of mildly pathogenic or asymptomatic infections, encountered at varying frequency in a wide range of marine and freshwater species [11,12]. While the prevalence in natural populations can be high, the infections are usually well controlled by the host’s antibodies [13]. Fish trypanosomes thus mostly served as a suitable model in studies of the immune response of fish to unicellular parasites [14], and their motility in a host’s bloodstream [15]. Until recently, they were only rarely associated with pathogenicity and economic loss [12,16]. However, there is a growing number of reports concerning severe fish trypanosomiasis outbreaks in several commercially farmed fish species. Here, we summarize the situation in China, where these parasites recently emerged as a growing threat to this rapidly expanding industry that currently produces over 27 million tons of fish annually [17].

## Species identification and pathogenic outbreaks of freshwater fish trypanosomes

It was in the blood of trout (*Salmo trutta*) from Switzerland where the first ever trypanosome was observed by Gabriel Valentin in 1841. The earliest documented record of fish trypanosomes in China is more recent and occurred at a low prevalence and low parasitemia in the blood of black carp (*Mylopharyngodon piceus*) [18]. Subsequently, trypanosomes were reported from various freshwater fish in China, yet they were not considered a threat to aquacultures, in agreement with the infections observed in wild fish populations being typically subclinical or asymptomatic [11,13,16].

The long-standing morphology-based “one-host-one-parasite” paradigm that led to the description of numerous trypanosome species in fish was rejected based on their polymorphic DNA sequences [19,20]. Critical evaluation of the data revealed that in the absence of whole-genome sequences, combined with insufficient biological differences, all species described so far from freshwater fish in China should be synonymized as *Trypanosoma carassii* [21].

**Fig 1:**
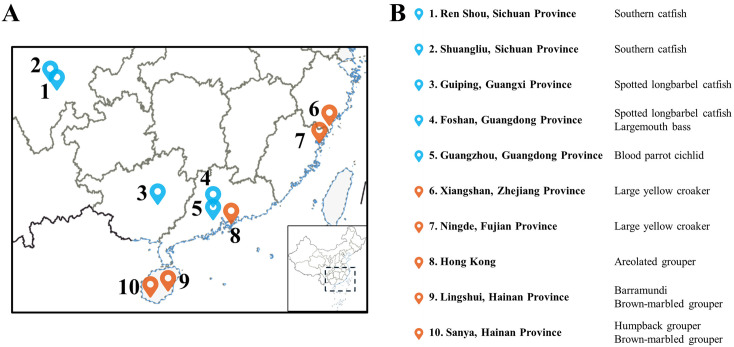
Geographic distribution and host species of fish trypanosomiasis outbreaks in aquacultures in Southern China. The left panel shows a map with outbreak locations in freshwater (blue label) and marine (orange label) aquacultures. Coastlines are shown in blue and national borders in black. An inset map in the lower right corner shows the full geographic extent of China (The base map is using data from the Aliyun Atlas platform (https://datav.aliyun.com/portal/school/atlas/area_selector). The right panel summarizes each outbreak location and the parasitized fish species.

Freshwater fish trypanosomiasis has emerged as a growing problem in aquaculture systems in China since the late 1990s (Fig 1 and S1 Table). The first recorded large-scale outbreak occurred in the Sichuan Province, where cages of cultured large-mouth catfish (*Silurus meridionalis*) experienced 30% mortality [22]. Another outbreak in the same province affected farmed southern catfish, with daily mortality reaching 4% [23]. Similar cases were reported from the Pearl River Basin, where mortality of farmed spotted long-barbel catfish (*Hemibagrus guttatus*) reached 80–100% in ponds, each stocked with approximately 20,000 fish, with up to 500 deaths per day [24]. Another economically valuable fish, the large-mouth bass (*Micropterus salmoides*), widely farmed across southern China, was subject to trypanosomiasis outbreaks in Guangdong Province [25,26]. Moreover, *T. carassii* caused 28% mortality in farmed ornamental hybrid parrot cichlids (*Vieja melanura × Amphilophus citrinellus*) [27]. Finally, trypanosome infections have also been observed in other cultured species, such as tilapia (*Oreochromis niloticus*) and blotched snakehead (*Channa maculata*). These parasites were also identified as *T. carassii* [21], suggesting that in China, the actual host range and prevalence of this pathogen may be much broader than currently documented.

## Pathogenic outbreaks and species identification of marine fish trypanosomes

Trypanosome infections of cultured marine fish reported in China invariably resulted in outbreaks, with the first incidence documented in cultured areolate grouper (*Epinephelus areolatus*) in Hong Kong, where mortality reached about 25% [28]. The population of caged, brown-marbled grouper (*Epinephelus fuscoguttatus*) in Hainan Province suffered a ~ 40% loss, caused by a newly described *Trypanosoma epinepheli* [29]. In Sanya, another outbreak of trypanosomiasis caused by this species involved both brown-marbled grouper and humpback grouper (*Cromileptes altivelis*), resulting in severe mortality in marine cages [30]. Subsequent investigations showed that *T. epinepheli* also infects barramundi (*Lates calcarifer*) cultured in coastal cages in the South China Sea, with daily mortality rates of 0.5-0.8% and cumulative losses reaching about 35% [31].

**Fig 2:**
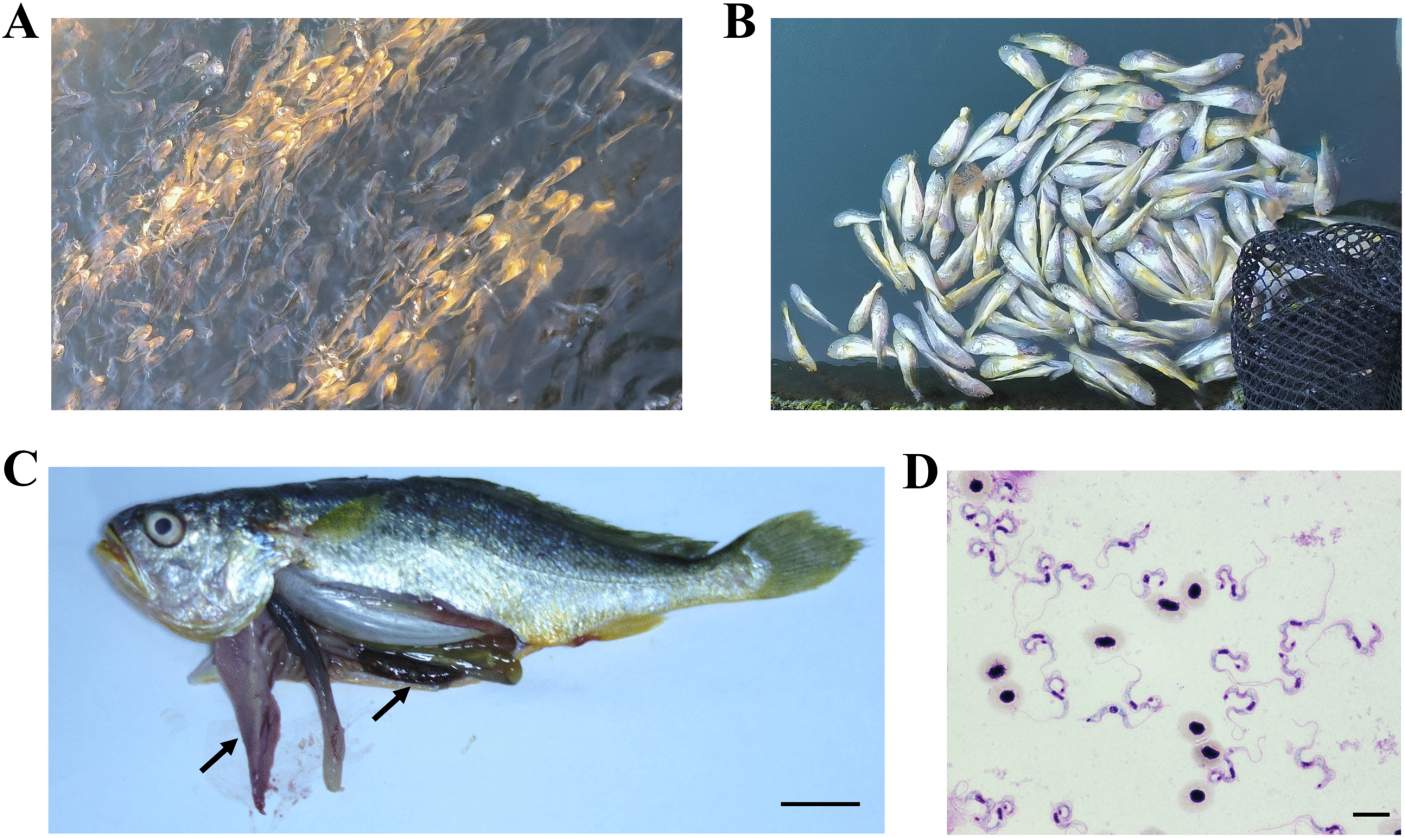
Representative clinical and pathological features of large yellow croaker (Larimichthys crocea) infected with *Trypanosoma carassii.* **(A)** High-density cage culture at a coastal mariculture farm in Ningde, Fujian Province. **(B)** Diseased and moribund fish showing abnormal swimming behavior and loss of equilibrium. **(C)** Gross pathology of infected fish displaying pale and enlarged liver and dark, swollen spleen. Scale bar, 2 cm. **(D)** Giemsa-stained blood smear showing numerous trypomastigotes (approximately 1 × 10⁹ parasites/mL). Scale bar, 10 μm. Photographer: Ju-feng Wang.

Perhaps the most dramatic losses that reached up to 60% mortality have been recorded in farmed large yellow croaker (*Larimichthys crocea*) cultured in Fujian and Zhejiang provinces (Fig 2) [32,33]. Morphological and molecular analyses confirmed the appurtenance of the causative agent to *T. carassii* [34], which was so far associated only with freshwater fish species [11,21]. This finding serves to blur the boundary between the infectivity of freshwater and marine fish trypanosomes [34], which is further supported by experimental infections showing that tilapia is susceptible to both *T. carassii* and *T. epinepheli* [35].

## How do trypanosomes cause disease and mortality in fish?

The clinical signs of fish trypanosomiasis are highly variable and influenced by multiple factors, such as host species and environmental conditions. In traditional low-intensity fish polycultures within semi-natural pond milieu, mild and chronic infections are characteristic, while a patent parasitaemia is rare [11] (J.L., unpubl. data). However, under intensive conditions with fish exposed to high stocking density, now dominating Chinese aquacultures, trypanosomes tend to cause noticeable morbidity and mortality (Fig 2 and S1 Table). The increased prevalence observed under high-density aquaculture conditions likely reflects multiple interacting factors. Fish reared at high stocking densities frequently experience chronic physiological stress and suboptimal health conditions, which may compromise immune competence. In addition, dense populations facilitate transmission by increasing vector biting frequency and contact rates among fish. Mechanical skin damage caused by crowding may potentially allow direct transmission *via* mucus or lesion contact, although this possibility remains unconfirmed.

During infection, affected fish commonly exhibit behavioral abnormalities, such as reduced appetite, lethargy, anorexia, erratic swimming, and/or surface floating just before death (Fig 2B) [26,27,29,31]. Gross pathological examinations typically include pale gills, marked splenomegaly caused by intense hemolysis, and congestion (Fig 2C). The liver is frequently enlarged, soft, or discolored, and the spleen often appears dark brown to black. Histopathology revealed systemic multi-organ damage, with the most severe lesions observed in the spleen, liver, kidney, and gill tissues. Numerous trypanosomes are frequently found within blood vessels and capillaries of the gill, liver, spleen, kidney, and occasionally the brain and myocardium. In heavily infected fish, these cumulative lesions result in anemia and metabolic exhaustion, ultimately leading to death. Indeed, experimentally infected fish commonly evince surface gasping, uncoordinated swimming, anorexia, anemia, and hepatosplenic alterations. Although mortality is consistently associated with high parasitemia, the contribution of secondary bacterial or viral co-infections cannot be excluded.

## What is the vector of fish trypanosomes?

The life cycle of fish trypanosomes is long known to be digenetic, involving both fish hosts and invertebrate bloodsucking vectors, invariably leeches belonging to several genera. Leydig first observed flagellated protozoans in leeches in 1857, while Doflein predicted their vectorial capacity for fish hemoflagellates already in 1901. Subsequently, the life cycle was experimentally confirmed using different freshwater and marine fish and leeches in Europe, and North and South America [36–39].

It is therefore surprising that among the numerous fish trypanosomiasis outbreaks recently reported in China, only one case noted leeches attached to fish fins and gills [29]. The prominent absence of leeches in cages and ponds harboring heavily infected fish motivated experiments that aim to monitor alternative ways of transmission of *T. carassii*, including those *via* other invertebrate vectors, through mucus exchange, open wounds, or by direct contact between infected and uninfected large-mouth bass (D.-H.L., unpubl. data). Thus, identifying the routes of transmission of trypanosomes in Chinese marine and freshwater aquacultures is an urgent priority.

## How is fish trypanosomiasis diagnosed in aquaculture settings?

The diagnosis of fish trypanosomiasis relies on microscopic and molecular methods. The most basic approach is a microscopic examination of fresh wet mounts from infected fish, allowing quick visualization of motile trypanosomes under light microscope, which can be further complemented by Giemsa-stained blood smears to visualize the single flagellum and characteristic, dot-shaped kinetoplast. While simple and inexpensive, these methods show poor sensitivity during early and chronic infections characterized by low parasitemia [40]. Several reports of mixed infections have been based on morphological examination, and rarely also via sequencing [41]. PCR-based assays such as conventional and quantitative PCR targeting the 18S rDNA or ITS genes are now widely used. These methods may offer high sensitivity and specificity, enabling both species identification and parasite quantification, and are especially valuable for subclinical or mixed infections [42]. In recent years, isothermal amplification techniques-including loop-mediated isothermal amplification (LAMP) and multiple cross displacement amplification (MIRA) have emerged as promising tools for rapid on-site diagnosis [43,44]. In particular, these assays may also be adapted for methods utilizing environmental DNA templates. These methods generally require relatively simple equipment and can provide rapid and good diagnostic performance under both laboratory and field conditions, suggesting their potential utility for field surveillance and early outbreak detection in aquaculture systems.

## How can we better control fish trypanosomiasis?

China hosts the world’s largest and most intensive aquaculture sector, often characterized by high stocking densities and rapid production cycles that likely facilitate parasite transmission and increase host stress. For these reasons, and also due to the growing attention to fish health, most trypanosome outbreak reports come from China. The most severe case in recent years involved large-scale outbreaks in large yellow croaker (Fig 2), resulting in substantial economic losses. Fishermen attempted to control the disease using common aquatic drugs as well as veterinary medicines used for mammalian trypanosomiasis, but all attempts failed (D.-H.L., unpubl. data). Consequently, fish from the trypanosome-infected farms had to be expedited for sale in order to minimize economic losses.

To inform preventive measures, the main route of transmission must be established. Moreover, a high-quality whole-genome assembly and comprehensive genomic analysis of *T. carassii* and other fish trypanosomes are urgently needed. Such genomic resources will improve our understanding of their biology, host-parasite interactions, and immune evasion mechanisms, allowing the design and development of vaccines and therapeutics. For instance, GP63 and trans-sialidase have been predicted as major surface coats in these fish trypanosomes [45], thus representing a promising target. Finally, more research is needed to explore the ecological drivers of emergence for these parasites under intensive aquaculture conditions, including the effects of temperature, salinity, and stocking density. Establishing integrated surveillance systems that combine parasitological, molecular, and ecological monitoring will be key to predicting outbreaks and mitigating their impact on China’s rapidly expanding aquaculture industry.

## Supporting information

S1 Table Reported outbreaks of fish trypanosomiasis in cultured fish in China. (DOCX)
