# Supplementary material for: Trypanosomes of fish as an emerging threat to aquaculture systems in China
Source: PLoS Pathog. 2026 Apr 28;22(4):e1014129. doi: 10.1371/journal.ppat.1014129 (PMC13123945; doi:10.1371/journal.ppat.1014129)
Supplement: S1 Table — (DOCX) [file ppat.1014129.s001.docx]

S1 Table Reported outbreaks of fish trypanosomiasis in cultured fish in China

| **Location** | **Host species** | **Environment** | **Outbreak information** | **Year** | **Ref.** |
| --- | --- | --- | --- | --- | --- |
| Ren Shou, Sichuan Province | *Silurus meridionalis* | freshwater | 1463/4800 fish, ~30% mortality | 1997 | [1] |
| Shuangliu, Sichuan Province | *Silurus meridionalis* | freshwater | 3-4% mortality per day during peak outbreak | 2011 | [2] |
| Guiping, Guangxi Province | *Mystus guttatus* | freshwater | Not reported | 2008 | [3] |
| Foshan, Guangdong Province | *Mystus guttatus* | freshwater | ~500 fish per day during peak outbreak | 2007 | [3] |
| Foshan, Guangdong Province | *Micropterus salmoides* | freshwater | 600-700 fish per day during peak outbreak | 2016 | [4] |
| Foshan, Guangdong Province | *Micropterus salmoides* | freshwater | 0.2-0.5% mortality per day | 2018 | [5] |
| Guangzhou, Guangdong Province | blood parrot cichlid | freshwater | ~1% mortality per day in peak outbreaks, ~28% total mortality | 2012-2022 | [6] |
| Hong Kong | *Epinephelus areolatus* | marine | ~25% mortality | 2002 | [7] |
| Lingshui, Hainan Province | *Epinephelus fuscoguttatus* | marine | ~40 % mortality | 2010 | [8] |
| Sanya, Hainan Province | *Cromileptes altivelis*  *Epinephelus fuscoguttatus* | marine  marine | Not reported | 2012 | [9] |
| Lingshui, Hainan Province | *Lates calcarifer* | marine | Not reported | 2017 | [10] |
| Ningde, Fujian Province | *Larimichthys crocea* | marine | ~1% mortality per day; ~70% total mortality | 2023 | [11] |
| Xiangshan, Zhejiang Province | *Larimichthys crocea* | marine | mortality (>100 kg fish per day) | 2024 | [12] |
